# Human VAMP3 Suppresses or Negatively Regulates Bax Induced Apoptosis in Yeast

**DOI:** 10.3390/biomedicines9010095

**Published:** 2021-01-19

**Authors:** Damilare D. Akintade, Bhabatosh Chaudhuri

**Affiliations:** 1 School of Life Sciences, Medical School, University of Nottingham, Nottingham NG7 2UH, UK; 2 Leicester School of Pharmacy, De Montfort University, Leicester LE1 9BH, UK; BChaudhuri@dmu.ac.uk

**Keywords:** human VAMP3, apoptosis, VAMP3 rescue Bax, Yeast and VAMP3, yeast apoptosis

## Abstract

Apoptosis is an essential process that is regulated genetically and could lead to a serious disease condition if not well controlled. Bax is one of the main proapoptotic proteins and actively involved in programmed cell death. It has been suggested that Bax induced apoptosis in yeast could be obstructed by enhancing vesicular membrane trafficking. Plasma membrane proteins and lipid oxidation were reduced by a vesicle-associated membrane protein (VAMP) when expressed in yeast, suggesting its potential role in repairing membranes. Membrane integrity is crucial, as the loss of membrane integrity will result in the leakage of ions from mitochondria, and ultimately cell death due to overproduction of reactive oxygen species (ROS). Expression of *Arabidopsis*’ VAMP has been linked to antiapoptosis activity. Since plant VAMP has been associated with antiapoptotic activities, this study investigates the possible participation of human VAMP3 in blocking human Bax mediated apoptosis. Some novel genes were identified to rescue Bax’s proapoptotic effects, in a yeast-based human hippocampal cDNA library screen. VAMP3 (a gene code for proteins involved in protein secretion) gene was chosen for further study to confirm its role in inhibiting apoptosis. VAMP3 was coexpressed with a chromosomally integrated Bax gene expression cassette driven by the GAL1 promoter. The antiapoptotic proteins of the Bcl-2 family (Bcl xL) were known to negate the proapoptotic properties of Bax. However, the new gene (VAMP3) results show that novel antiapoptotic proteins can be identified using a yeast-based assay. The findings presented here show that human VAMP3 protein has antiapoptotic property and could abrogate Bax induced apoptosis (cell death).

## 1. Introduction

The initiation of apoptosis results in destruction cells, and eventually, cell death [1]. VAMPs (vesicle-associated membrane proteins) form complexes with synaptosome associated protein (SNAP-25), proteins that are found in target membranes to facilitate vesicular trafficking during the processes of secretion and endocytosis [2]. It has been shown that Bax induced apoptosis in yeast can be obstructed through the enhancement of vesicular membrane trafficking [3], although yeast does not have endogenous Bax when Bax is ectopically expressed in yeast, it kills cells [4]. When VAMP is coexpressed with Bax, it prevents it from killing. Such membrane trafficking includes transferrin receptor recycling, phagocytosis, and pinocytosis [5]. VAMP3 was implicated in acting as a negative regulator to the replication of Leishmania amazonensis by limiting cholesterol availability [6]. Additionally, miR-124/VAMP3 has been linked in surgery where it induces microglial activation, and it was reported that postoperative disorders mediated by microglial activation could have a potential therapeutic strategy by targeting miR-124/VAMP3 [7].

Necrosis and apoptosis have been identified in yeast [8]. Apart from yeast, programmed cell death has also been observed in other unicellular organisms such as *Trypanosoma brucei, Trypanosoma cruzi, Tetrahymena thermophila* and *Dictyostelium discoideum*, these organisms display some features of apoptosis [9]. When considering the evolutionary tree, these organisms are further distant to mammals compared to yeast. This would imply that the apoptotic pathway is conserved [10]. Plants also exhibit apoptosis [11].

VAMP3, a tetanus neurotoxin-sensitive SNARE (soluble N-ethylmaleimide-sensitive factor attachment protein receptor), facilitates fusion with Golgi and endocytic recycling compartment after it segregates into tubular membranes. It regulates the recycling of the transferrin receptor, its ligand transferrin and integrins to the plasma membrane. It is also involved in granule transport in platelets [12]. VAMP3 is a vesicular SNARE protein that resides in endosome-derived transport vesicles and recycling endosomes [13]. Intracellular membrane fusion is mediated by the paring of v-SNARE (vesicular SNARE) and t-SNARE (target membrane SANRE) [14]. The three families of SNARE proteins include (a) target membrane localised SNAPs, (b) VAMPs, and (c) syntaxins (membrane proteins located on target membranes). They network to form SNARE complexes through its SNARE domains [13]. VAMP3 is generally expressed in all non-neuronal tissues, and it binds syntaxin 4 to tie vesicular cargo to the membrane. It recycles receptors via recycling endosomes [13]. Interruption of VAMP3 could result in an abnormal location of low-density lipoprotein receptor and transferrin [13]. VAMP3 is therefore crucial in effective intracellular transport, which is essential for the proper functioning of cells.

The VAMP isolated from *Arabidopsis* is close to rat VAMP7 (64% similarity) [3]. Plasma membrane proteins and lipid oxidation were reduced by VAMP when expressed in yeast, suggesting its potential role in repairing membranes. Membrane integrity is crucial, as a loss of membrane integrity will result in leakage of ions, and ultimately cell death [3,15]. Intracellular membrane fusion machinery has SNARE proteins as its core [14]. Studies have also shown that VAMP3 is essential for the movement of cells via hb-1 integrin trafficking. Disruption of VAMP3 leads to abridged movement rate in injured epithelial cells [13]. Bax stimulates apoptosis while Bcl-2 subdues apoptosis. Expression of *Arabidopsis’* VAMP obstructed both Bax and hydrogen peroxide triggered apoptosis in yeast by acting downstream of the oxidative burst induced by these two reagents [3].

Some novel genes were identified to rescue Bax’s proapoptotic effects, in a yeast-based human hippocampal cDNA library screen, as previously described and patented (publication number: 20090258794) (Bhabatosh Chaudhuri, 2009) [16]. VAMP3 (a gene code for proteins involved in protein secretion) gene was chosen in this study for further investigation to confirm its role in the inhibition of apoptosis. Since plant VAMP has been linked to antiapoptotic activities, this study investigates the possible participation of human VAMP3 in blocking human Bax mediated apoptosis. Yeast strain harbouring one copy of chromosomally integrated Myc-tagged Bax gene (Supplementary Information Figure S1) (Bax(LEU2) was transformed with an episomal 2-micron plasmid that encodes HA-tagged VAMP3 gene (Supplementary Information Figure S2). The resultant strain Bax(LEU2): VAMP3 would allow coexpression of Bax and VAMP3 in yeast cells.

Levine at al. 2001 reported that VAMP in *Arabidopsis* (a plant) has an antiapoptotic property [3]. This study examined the human VAMP3 antiapoptotic activity. The highest homology of the isolated sequence (NAtVAMP) from Levine et al., 2001, was in the VAMP7 gene from rat (41% identity and 64% similarity). Many fundamental hallmarks might be conserved among animal and plant cells via evolution. There are different VAMPs in human, which has not been linked to apoptosis. VAMP3 is relevant to human disease progression and treatment. It also plays an essential role in cell homeostasis, morphogenesis, and pathogen defence.

## 2. Materials and Methods

### 2.1. Yeast Strains

The yeast strain W303-1A Mata (ATCC #208352) is auxotrophic for adenine, histidine, leucine, tryptophan, and uracil due to a mutation in genes *ADE2*, *HIS3*, *LEU2*, *TRP1,* and *URA3*. New yeast strains were derived by transforming integrative plasmids expressing Bax-myc tagged from the *GAL1* promoter or episomal plasmid expressing VAMP3 -HA-tagged, or Bcl xL-HA tagged gene on PGK promoter (Supporting Information, Figures S1–S3).

### 2.2. Yeast Transformation

Plasmids bearing Bax gene expression cassettes under the galactose-inducible *GAL1* promoter (*GAL1*p; see Supporting Information, Figure S1) were used for genomic integration at the *LEU2* chromosomal loci of the yeast strain to yield strains that contain one copy of Bax. Additionally, an episomal plasmid bearing VAMP3 or Bcl xL gene expression cassettes on a PGK1 promoter. The integrative transformation was carried out using a published protocol [17].

### 2.3. Detection of Dead Cells with Phloxine B Dye

Cell death was assessed by staining cells with the red dye Phloxine B (Sigma, Dorset, UK. P4030-25G) [18]. Phloxine B (Sigma, P-4030-25G) was added to both test and control yeast cells to a final concentration of 5 μg/mL and incubated at 30 °C for 30 min in the dark. Staining experiments were performed as published earlier [19].

### 2.4. Detection of Reactive Oxygen Species (ROS)

Reactive oxygen species (ROS) was measured using the AAT Bioquest Fluorimetric Intracellular Total ROS Activity Assay Kit (#22901, Sunnyvale, CA, USA). Experiments were performed as published earlier [19].

### 2.5. Quantifying Mitochondrial Membrane Potential (MMP) with the JC-10 Dye

AAT Bioquest JC-10 Mitochondrial Membrane Potential Assay kit (22800, Sunnyvale, CA, USA) was used for measuring mitochondrial membrane potential. The kit uses JC-10 dye. Experiments were conducted as per the published protocol [19].

### 2.6. Staining with Hoechst Dye for Monitoring Live Cells

Hoechst 33,258 (Thermo Fisher Scientific; #H21491, Loughborough, UK) is a widely used nucleic acid stain to detect live cells. Staining with the dye was performed as described earlier [19].

### 2.7. Assessing Nuclear DNA Fragmentation via the TUNEL Assay

DNA fragmentation was detected by terminal deoxynucleotidyl transferase (TdT)-facilitated dUTP nick end labelling (TUNEL). The AAT Bioquest TUNEL Apoptosis Assay kit (#22844, Sunnyvale, CA, USA) was used to detect nuclear DNA fragmentation (NDF). The assays were performed as described earlier [19].

### 2.8. Western Blotting

Western blotting was carried out using standard protocols [20], using primary antibodies specific to c-Myc (Thermo Scientific, #MA 1-980, Loughborough, UK) and HA-tag (Proteintech, #51064-2-AP) or β-actin (Proteintech; #60008-1-Ig; Manchester, UK).

## 3. Results and Discussion

### Coexpression of Human VAMP3 from PGK1 Promoter and Bax from GAL1p in Yeast along with Bcl xL on PGKp

**Figure 1:**
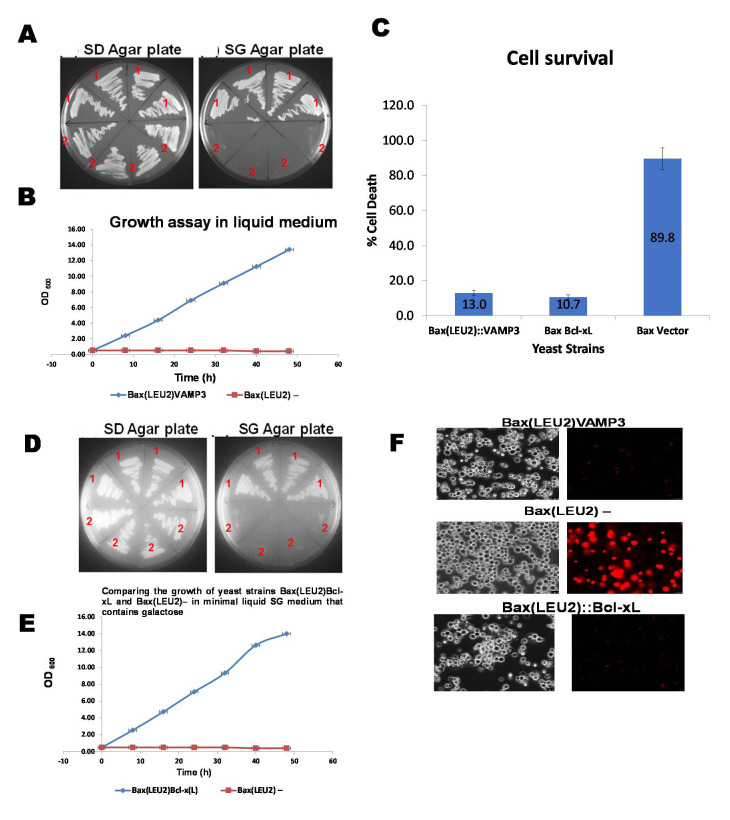
The growth assays on the solid and liquid media and death assay (Phloxin B assay). (**A**,**D**) Growth of yeast strains in solid agar plates over 72 h, in the glucose-containing minimal medium (S.D.), and galactose containing minimal medium (S.G.). The 4 strains on the upper half of the two plates are Bax(LEU2)VAMP3 (**A**) and Bax(LEU2)Bcl-xL (**D**) transformants (1), containing an episomal 2µ-plasmid plasmid that encodes the HA-tagged VAMP3 **(A)** and Bcl-xL (**D**) genes and a Bax expression cassette integrated at the LEU2 locus. The 4 strains, in the lower half of the plates (**A**,**D**), are Bax(LEU2)–(2) transformants that contain the Bax expression cassette and an empty plasmid. (**B**,**E**) Growth of yeast cells, Bax + VAMP3 (**B**) and Bax + Bcl-xL (**E**), in the minimal liquid medium containing galactose, throughout 48 h along with the control strain. A two-tailed paired sample *t*-test shows, statistically, that there was a significant difference (*p* < 0.05). (**C**) The percentage of cell death in strains Bax + VAMP3, Bax + Bcl-xL and control Bax(LEU2)–, after growth in galactose for 48 h. The data represent the mean ± S.D. of three independent experiments. Dead cells stained with phloxine B are shown in (**F**) The images on the left a representative of cells under fluorescent light, showing death cells (red). A two way ANOVA test shows that there was a significant difference (*p* < 0.05) in cell death between the control strain and each of Bax + VAMP3 and Bax + Bcl-xL strains, but there was no significant difference (*p* > 0.05) between Bax + VAMP3 and Bax + Bcl-xL.

A plasmid encoding the human Bax gene (Bax-α) was integrated into the yeast strain’s LEU2 chromosomal locus. The Bax gene is under the control of the GAL1 promoter. This results to the yeast strain Bax(LEU2), which has also been referred to as “Bax(LEU2)” or just “Bax.”, The Bax(LEU2) yeast strain was then transformed with an episomal plasmid bearing VAMP3 or Bcl xL gene (as the positive control) separately. VAMP3 and Bcl xL were coexpressed with the Bax gene in the resultant strains Bax(LEU2)VAMP3 and Bax(LEU2)Bcl-xL shown in Figure 1. Figure 1A shows the growth of Bax(LEU2)VAMP3 and Bax(LEU2) yeast strains on the minimal solid agar medium and Figure 1D shows the growth of Bax(LEU2)Bcl-xL and Bax(LEU2) yeast strains on minimal solid medium. The negative control strain (Bax(LEU2)) did not grow on the solid medium, while Bax(LEU2)VAMP3 and Bax(LEU2)Bcl-xL grew on the solid agar medium. Figure 1B shows the growth of Bax(LEU2)VAMP3 and Bax(LEU2) yeast strains in a minimal liquid medium and Figure 1E shows the growth of Bax(LEU2)Bcl-xL and Bax(LEU2) yeast strains in a minimal liquid medium. Similarly, The negative control strain (Bax(LEU2)) did not grow on the liquid medium, while Bax(LEU2)VAMP3 and Bax(LEU2)Bcl-xL grew on the liquid medium. Figure 1C shows the percentage cell death of yeast strains Bax(LEU2)Bcl-xL, Bax(LEU2)VAMP3 and Bax(LEU2) after Phloxin B staining, while Figure 1F is a representative image of cells after Phloxin B staining. The negative control strain (Bax(LEU2)) had the highest cell death percentage than the other two strains.

**Figure 2:**
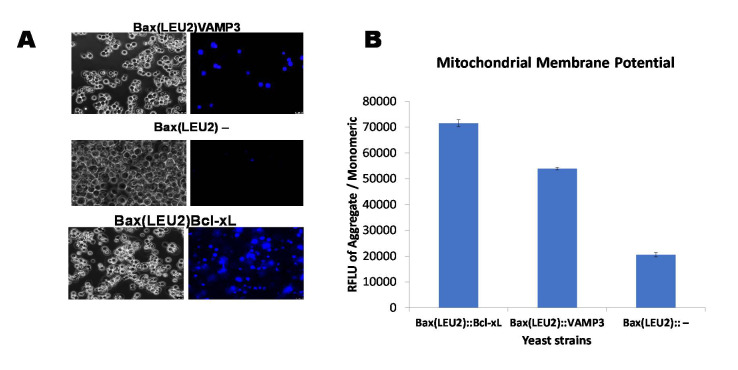
Live assay (Hoechst 33,342 dye) and measurement of the mitochondrial membrane potential. (**A**) Visualisation of yeast cells that coexpress Bax and VAMP3 and Bcl-xL or Bax alone, after staining with Hoechst 33,342 dye. The images on the left are representative of cells under fluorescent light, showing live cells (blue). (**B**) Quantification of the mitochondrial membrane potential of yeast strains, Bax(LEU2)VAMP3, which coexpressed Bax and VAMP3, Bax(LEU2)Bcl xL, which coexpressed Bax and Bcl xL, and Bax(LEU2), which expresses Bax alone, using a fluorescent plate reader. A two way ANOVA test shows that there was a significant difference (*p* < 0.05) between the control strain and each of Bax + VAMP3 and Bax + Bcl-xL, but there was a significant difference (*p* < 0.05) between Bax + VAMP3 and Bax + Bcl-xL. The data represent the mean ± S.D. of three independent experiments. RFLU—Relative Fluorescence Unit.

Figure 2A is a representative image of Hoechst 33,342 dye staining of live cells in yeast strains Bax(LEU2)Bcl-xL, Bax(LEU2)VAMP3 and Bax(LEU2), the negative control strains show the least sign of live cells. Figure 2B show the measurement of mitochondria potential in the three yeast strains Bax(LEU2)Bcl-xL, Bax(LEU2)VAMP3 and Bax(LEU2), the positive control (Bax(LEU2)Bcl-xL) has the highest mitochondrial membrane potential followed by Bax(LEU2)VAMP3.

**Figure 3:**
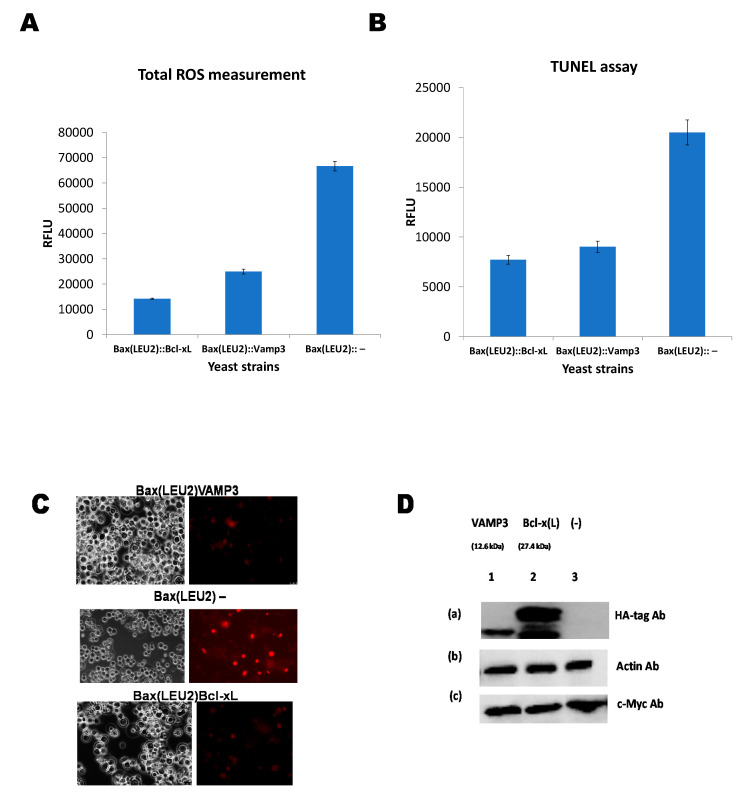
Reactive oxygen species (ROS) measurement, terminal deoxynucleotidyl transferase (TdT)-facilitated dUTP nick end labelling (TUNEL) assay, and Western blotting. (**A**) Measurement of ROS produced in the yeast strains Bax(LEU2) + VAMP3, Bax(LEU2) + Bcl xL, and Bax(LEU2) –. A two way ANOVA test shows that there was a significant difference (*p* < 0.05) between the control strain and each of Bax + VAMP3 and Bax + Bcl-xL, but there was no significant difference (*p* > 0.05) between Bax + VAMP3 and Bax + Bcl-xL. The data represent the mean ± S.D. of three independent experiments. (**B**) TUNEL assay comparing the nuclear DNA fragmentations in yeast strains Bax(LEU2)VAMP3, Bax(LEU2)Bcl xL, and Bax(LEU2). The cells Bax(LEU2)VAMP3 and Bax(LEU2)Bcl xL have lower nuclear DNA fragmentation compared to Bax(LEU2)–cells. A two way ANOVA test shows that there was a significant difference (*p* < 0.05) between the control strain and each of Bax + VAMP3 and Bax + Bcl-xL, but there was no significant difference (*p* > 0.05) between Bax + VAMP3 and Bax + Bcl-xL. The data represent the mean ± S.D. of three independent experiments. (**C**) Represent the microscopic image of cells after tunnel assay, Bax(LEU2)VAMP3 and Bax(LEU2)Bcl xL. The left images are representative of cells under fluorescent light, showing dead cells (red) (high nuclear fragmentation)**.** (**D**) Western blot to monitor the presence of Bax in yeast cells that express only Bax and in cells that coexpress Bax together with VAMP3 and, Bcl-xL. Of total cellular proteins, in cell lysates, 10 µg were loaded on to each lane and the blot was probed with an antibody that recognises the c-Myc-tag, Actin, and HA-tag. RFLU—Relative Fluorescence Unit.

Figure 3A shows the ROS measurement in yeast strains Bax(LEU2)Bcl-xL, Bax(LEU2)VAMP3, and Bax(LEU2). The negative control strain produces the highest ROS compared to the other strains. Similarly, Figure 3B shows that the negative control strain has higher nuclear DNA fragmentation from the TUNNEL assay than the other strains. Figure 3C is a representative image of the TUNNEL assay. Figure 3D shows the Western blot of the various protein from the three yeast strains Bax(LEU2)Bcl-xL, Bax(LEU2)VAMP3, and Bax(LEU2).

The results show that the four strains on the top half of the S.G. agar plates (Figure 1A,D) grew on galactose. In comparison, the four strains on the bottom half did not grow, indicating that proapoptotic Bax is toxic to yeast, but its toxicity can be rescued in yeast by the antiapoptotic Bcl-xL and VAMP3 proteins. Bcl-xL is a widely known antiapoptotic protein of the Bcl-2 family. Bcl-xL has been used, as positive controls, to confirm that these known antagonists of human Bax can overcome Bax’s toxic effects in yeast.

A homolog of synaptobrevins or vertebrate synaptic vesicle-associated membrane proteins (VAMPs) in yeast *Saccharomyces cerevisiae* is SNC1. It can suppress mRNA CAP function loss in *Saccharomyces cerevisiae* strains having RAS2 (a guanine nucleotide-binding protein) allele activated [21]. It influences the starvation response and growth regulation. One hundred and seventeen (117) amino acids encode the SNC1 gene, which is very homologous to synaptobrevin/VAMP family members. They are believed to be linked to synaptic vesicles targeting and fusion with the presynaptic membrane [21].

The growth curve in liquid culture (Figure 1B,E) and the growth of cells on solid agar plates (Figure 1A,D) indicate that cells that expressed VAMP3 and Bcl xL genes were rescued from Bax induced apoptosis. Cells without VAMP3 or Bcl xL but express Bax alone (control cells) did not show any growth. A death assay was then carried out to ascertain the levels of rescue from death. Phloxine B staining (Figure 1C,F) and Hoechst stain (Figure 2A) were employed to determine death and life levels, respectively, as shown in Figure 1C and Figure 2A. Significantly less cell death occurred in Bax(LEU2) VAMP3 cells, which coexpress Bax and VAMP3, likewise in Bax(LEU2) Bcl-xL compared to control cells Bax(LEU2), which express Bax alone, this suggests that human VAMP3 rescues yeast cells from Bax-mediated cell death. The Hoechst dye stains live cells by binding to nuclear DNA (binding readily to A-T rich regions). Cells that express Bax alone do not have any live cells, whereas cells coexpressing VAMP3 have many more live cells. The VAMP isolated from *Arabidopsis* is close to rat VAMP7 (64% similarity) [3].

Only the homodimers of Bax are proapoptotic. Antiapoptotic Bcl-2 proteins form heterodimers with Bax. It has been suggested that this heterodimerisation nullifies Bax’s toxic effects and thereby prevents Bax-mediated apoptosis. In some instances, binding of Bcl-2 to Bax is not enough to stop apoptosis. However, apoptosis induced by triggers other than Bax can be repressed by overexpression of Bcl-2 and Bcl-xL even in the absence of Bax [22]. The mitochondrial membrane potential is essential in the determination of cell function and viability [23]. It is the primary metabolic function that symbolises the harsh effects on the mitochondria during apoptosis; typical mitochondrial membrane potential is essential for mitochondrial protein and RNA synthesis [24]. The mitochondrial membrane potential in cells containing VAMP3 and Bcl-xL (Figure 2B) was significantly higher than in the control cells Bax(LEU2)−. Plasma membrane proteins and lipid oxidation were reduced by VAMP when expressed in yeast, suggesting its potential role in repairing membranes. Membrane integrity is crucial, as loss of membrane integrity will result in a leakage of ions, and ultimately cell death [3].

The typical secretory pathway helps deliver freshly produced lipids and proteins to the surface of the cell, which is vital for cell viability and growth, including secretion of extracellular matrix components, antibodies, and cytokines [25]. Many of these secreted proteins are transported from the endoplasmic reticulum via Golgi apparatus and then the cell surface where they fused with the plasma membrane (driven by SNARE proteins) [25]. VAMP3 and YKT6 depletion in mammalian cells was reported to cause a blockage in secretion [25]. This suggests that VAMP3 and YKT6 are involved in the late part of the secretory pathway.

Quantification of ROS produced from yeast strains expressing Bax and VAMP3 (Figure 3A) shows that the difference in ROS production was significant compared to the strain, which expresses Bax alone. The latter produced more ROS compared to the strain that coexpressed VAMP3. The results suggest that human VAMP3 protein had antiapoptotic properties, allowing rescuing proapoptotic protein Bax’s toxic effects in yeast. This result was similar to the strain expressing Bcl xL (Figure 3A). The build-up of ROS is a trademark of apoptotic cell death. Bax stimulates apoptosis in both human and yeast cells via the production of ROS [15]. Bax translocates from the cytosol to the mitochondria damaging the mitochondrial membrane during apoptosis. The TUNEL assay (Figure 3B,C) corroborated the findings, with lower DNA fragmentation for yeast strains Bax(LEU2) VAMP3 compared to Bax(LEU2)–, similarly, Bcl xL has lower DNA fragmentation.

Expression of *Arabidopsis’* VAMP obstructed both Bax and hydrogen peroxide triggered apoptosis in yeast by acting downstream of the oxidative burst induced by these two reagents [3]. The Western blot in Figure 3D confirms the expression of the respective proteins. Though generally, Bcl xL seems to have higher rescue power than VAMP3, however, the differences are not significant. VAMP3, SNAP23 and syntaxin-13 are essential in the trafficking of matrix metalloproteinases throughout the degradation of extracellular matrix substrates [26]. Extracellular vesicles are membrane-derived vesicles. They are endogenous. They carry bioactive particles between the neurons and glia, supporting the survival neurons and the flexibility of the central nervous system, eventually impacting neurodegenerative disorders [27]. VAMP3 and syntaxin 6 have been linked to regulation of the fusion between Group A *Streptococcus* (GAS) containing autophagosome like vacuoles (GcAVs). Additionally, recycling endosomes (R.E.s) [28]. This has an impact on autophagy/macroautophagy and hence immunity.

## 4. Conclusions

VAMP3 has been linked with the regulation of some essential pathways in our system, which could involve the progression/treatment of some diseases. Apoptosis is vital to life, inappropriate activation of apoptosis could result in diseases, which include AIDS (acquired immunodeficiency syndrome), ischemic strokes, neurodegenerative disorders, autoimmune diseases, and oncogenesis. Plant VAMP was linked to antiapoptotic activities. On investigating the possibility of human VAMP3 in blocking Bax mediated apoptosis, the results in this study have demonstrated the antiapoptotic properties of VAMP3 protein involved in vesicular trafficking in the secretion pathway. Extracellular matrix cellular remodelling is a vital factor in several pathological and physiological processes; this remodelling relies on the secretion and trafficking of matrix metalloproteinases.

## Data Availability

Not applicable.

## Supplementary Materials

The following are available online at https://www.mdpi.com/2227-9059/9/1/95/s1, Figures S1–S3.
