# Supplementary material for: Human VAMP3 Suppresses or Negatively Regulates Bax Induced Apoptosis in Yeast
Source: Biomedicines. 2021 Jan 19;9(1):95. doi: 10.3390/biomedicines9010095 (PMC7835773; doi:10.3390/biomedicines9010095)
Supplement: Supplementary file 1 [file biomedicines-09-00095-s001.pdf]

Supporting information

Human VAMP3 Suppresses or Negatively Regulates Bax induced Apoptosis in Yeast

Damilare D. Akintade[a,b]* and Bhabatosh Chaudhuri[b]

[a] School of Life Sciences, Medical School, University of Nottingham. NG7 2UH, UK

[b] Leicester School of Pharmacy, De Montfort University, Leicester, LE1 9BH, UK

* Corresponding author

Dr Damilare Akintade

E-mail: Damilare.Akintade@nottingham.ac.uk

[a] Current address: School of Life Sciences, Medical School, University of Nottingham. NG7 2UH, UK

CONTENTS

SECTION 1. The plasmid construct bearing the human Bax-α gene and the resultant yeast strain.

SECTION 2. Plasmid constructs bearing the human vesicle-associated membrane proteins (VAMP3) gene and the resultant yeast strains.

SECTION 3. Plasmid constructs bearing the Bcl-xL gene and the resultant yeast strains.

<u>SECTION 1</u>: The plasmid construct bearing the human Bax-α gene and the resultant yeast strain

The human *Bax-α* (*Bax*) gene is under the control of the galactose-inducible *GAL1* promoter and was cloned in an integrating vector that bears the *LEU2* auxotrophic marker. The resultant plasmid was integrated into the *LEU2* chromosomal locus of the yeast strain W303 1A1. The map of the plasmid is shown below.

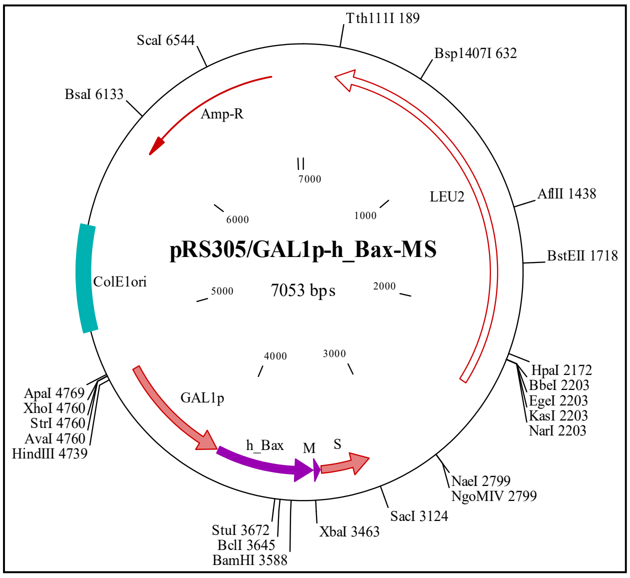

Figure S1. The plasmid map of pRS305/GAL1p-h_Bax-MS showing restriction sites that cut the plasmid only once. The human Bax gene contains, at the 3'-end, a sequence that codes for the *c*-myc tag so that expressed protein can be monitored with an antibody that recognises the *c*-myc epitope at the C-terminus of Bax protein.

SECTION 2: **Plasmid constructs bearing the human vesicle-associated membrane proteins (VAMP3) gene and the resultant yeast strains**

The human VAMP3 gene was cloned downstream of the PGK1 promoter in episomal plasmids bearing URA3 auxotrophic marker. The plasmids were then transformed into the yeast strains W303 1A1 carrying one copy of Bax gene. The plasmid map is shown below.

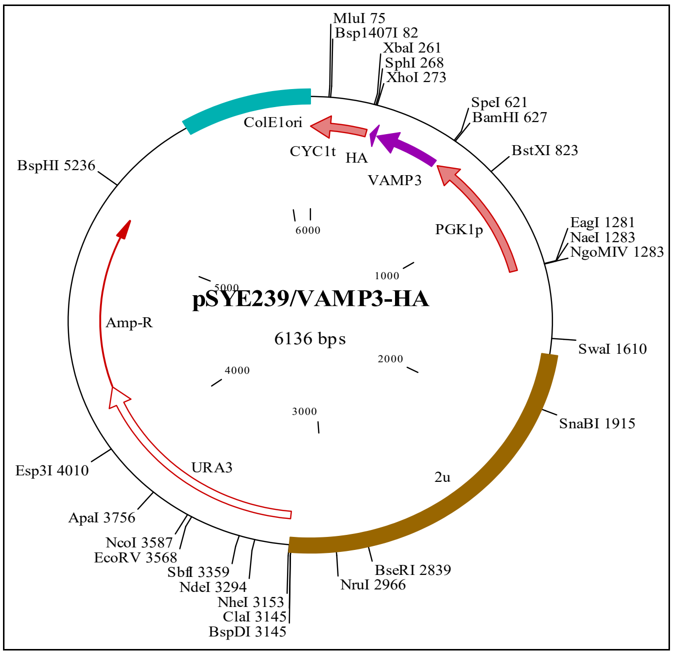

Figure S2. Plasmid map of pSYE239/VAMP3-HA, an episomal 2u-plasmid encoding HA-tagged VAMP3 gene. The restriction sites shown are the ones that cut the plasmid only once.

SECTION 3: **Plasmid constructs bearing the Bcl-xL gene and the resultant yeast strains**

The Bcl-xL gene was cloned downstream of the PGK1 promoter in episomal plasmids bearing URA3 auxotrophic marker. The plasmids were then transformed into the yeast strains W303 1A1 carrying one copy of Bax gene. The plasmid map is shown below.

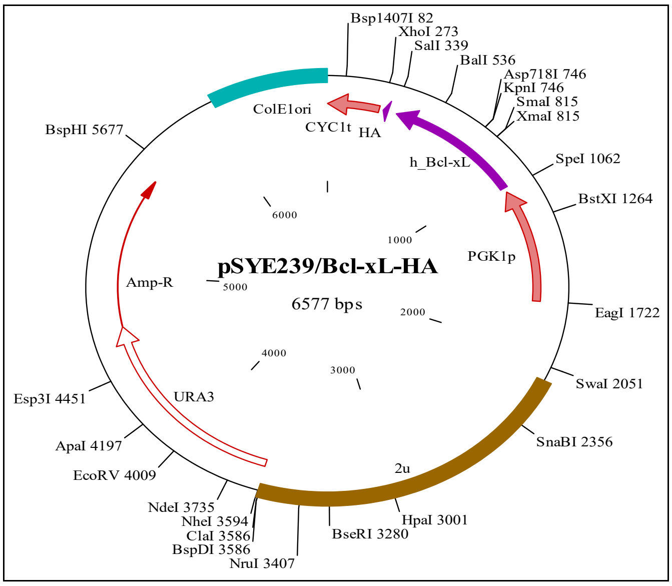

Figure S3. Plasmid map of pSYE239/Bcl-xL-HA, an episomal 2u-plasmid encoding HA-tagged Bcl-xL gene. The restriction sites shown are the ones that cut the plasmid only once.
